# Conjunctival Squamous Cell Carcinoma Masquerading as Orbital Cellulitis in a Patient With Relapsed Mixed Phenotype Acute Leukemia

**DOI:** 10.7759/cureus.113655

**Published:** 2026-07-30

**Authors:** Alireza Izadian Bidgoli, Alberto Gomez Usatorres, Alberto Gomez Veliz

**Affiliations:** 1 Internal Medicine, American University of the Caribbean School of Medicine, Cupecoy, SXM; 2 Internal Medicine, Jackson Memorial Hospital, Miami, USA; 3 Internal Medicine, Universidad Hispanoamerica, San Jose, CRI

**Keywords:** diagnostic challenge, immunocompromised host, mixed phenotype acute leukemia, orbital cellulitis, orbital squamous cell carcinoma

## Abstract

Conjunctival squamous cell carcinoma (SCC) rarely presents as orbital cellulitis, creating a significant diagnostic challenge in profoundly immunocompromised patients. We report the case of a 40-year-old man with relapsed mixed phenotype acute leukemia (MPAL) receiving prolonged leukemia-directed therapy who developed progressive unilateral periorbital swelling, pain, and imaging findings consistent with orbital cellulitis. Despite broad-spectrum antimicrobial therapy, serial computed tomography and magnetic resonance imaging demonstrated persistent superior orbital soft tissue thickening without meaningful clinical resolution. Because of the atypical clinical course, ophthalmologic biopsy was performed and demonstrated invasive moderately differentiated conjunctival SCC with lymphovascular invasion. Peripheral blood flow cytometry excluded lymphomatous involvement, confirming a second primary epithelial malignancy rather than leukemic infiltration. This case illustrates how prolonged leukemia-associated immunosuppression and intensive chemotherapy may obscure the diagnosis of secondary ocular surface malignancy by mimicking orbital infection. In patients with persistent orbital inflammation despite appropriate antimicrobial therapy, particularly those with relapsed hematologic malignancies and profound neutropenia, failure to improve should prompt early tissue biopsy, even when radiographic findings remain suggestive of orbital cellulitis, to avoid delays in definitive diagnosis and management.

## Introduction

Orbital cellulitis is an ophthalmologic emergency that can result in vision loss, intracranial extension, and other life-threatening complications if not promptly recognized and treated [1]. Consequently, patients presenting with orbital inflammation are often managed empirically for infection, particularly when imaging demonstrates soft tissue edema, abscess formation, or other inflammatory changes [1,2]. However, several noninfectious conditions, including inflammatory orbital disease, lymphoma, metastatic disease, and ocular surface squamous neoplasia (OSSN), may closely mimic orbital cellulitis clinically and radiographically, making accurate diagnosis particularly challenging [2].

Patients with mixed phenotype acute leukemia (MPAL) frequently require intensive chemotherapy and may develop prolonged immunosuppression, impaired immune surveillance, and chemotherapy-induced mucosal injury, predisposing them not only to opportunistic infections but also to secondary malignancies [3,4]. In this setting, persistent orbital lesions may represent infection, leukemic infiltration, treatment-related inflammatory changes, or a second primary malignancy, all of which may present with overlapping clinical and radiographic features [2-4].

OSSN encompasses a spectrum of epithelial malignancies ranging from conjunctival intraepithelial neoplasia to invasive squamous cell carcinoma (SCC) and represents the most common non-pigmented malignancy of the ocular surface [5,6]. Although conjunctival SCC typically presents as a localized ocular surface lesion, advanced disease may extend into adjacent orbital tissues and masquerade as orbital cellulitis, delaying definitive diagnosis [6,7].

We report the case of a patient with relapsed MPAL receiving ongoing leukemia-directed therapy who developed invasive conjunctival SCC presenting as presumed orbital cellulitis. This case emphasizes that persistent orbital inflammation despite appropriate antimicrobial therapy should prompt early tissue biopsy in profoundly immunocompromised patients, even when the initial clinical and radiographic findings strongly favor infection [2,7].

## Case presentation

Clinical presentation

A 40-year-old man with a history of MPAL receiving treatment with gilteritinib and venetoclax was transferred to our institution for further evaluation of progressive left periorbital swelling concerning for orbital cellulitis. Three days before presentation, he developed left eye pain accompanied by progressive eyelid swelling, serosanguineous drainage, photophobia, fever, and chills. He reported increasing difficulty opening the affected eye because of worsening eyelid edema but denied acute vision loss or diplopia.

Computed tomography of the orbits demonstrated diffuse left preseptal and lateral periorbital soft tissue edema with an ill-defined peripherally enhancing collection concerning for evolving phlegmon or abscess, together with enlargement of the left lacrimal gland. The patient was started on broad-spectrum intravenous antimicrobial therapy with cefepime, vancomycin, and metronidazole for presumed orbital cellulitis. Despite approximately two weeks of antimicrobial therapy, only partial clinical improvement was observed, prompting ophthalmology and oculoplastic surgery to pursue tissue biopsy for definitive diagnosis.

Initial assessment and physical examination

On presentation, the patient appeared chronically ill and cachectic but was alert and oriented without acute respiratory or hemodynamic compromise. Physical examination demonstrated profound chemotherapy-associated pancytopenia with a white blood cell count of 0.5 × 10³/µL, an absolute neutrophil count of 0 cells/µL, hemoglobin of 8.3 g/dL, and platelet count of 12 × 10³/µL.

Ophthalmologic examination revealed severe left periorbital edema with near-complete eyelid closure and serosanguineous drainage. Initial visual acuity measured 20/25 in the right eye and 20/40 in the left eye, with an intraocular pressure of 8 mmHg in the affected eye. Mild restriction of extraocular movements was present in the left eye secondary to marked periorbital swelling. Slit-lamp examination demonstrated conjunctival injection and chemosis with a corneal epithelial defect but no evidence of endophthalmitis. The left upper eyelid remained tense and indurated with persistent tenderness despite antimicrobial therapy. No afferent pupillary defect or optic nerve dysfunction was identified, and funduscopic examination demonstrated a flat, attached retina with a grossly normal optic nerve.

The remainder of the physical examination was unremarkable. Cardiopulmonary and abdominal examinations demonstrated no acute abnormalities. No generalized lymphadenopathy or focal neurologic deficits were identified. The absence of cranial nerve deficits, altered mental status, or bilateral ocular involvement made cavernous sinus thrombosis less likely at presentation.

Laboratory and diagnostic testing

Initial laboratory evaluation demonstrated profound pancytopenia consistent with relapsed MPAL and chemotherapy-induced marrow suppression during treatment with gilteritinib and venetoclax (Table 1). White blood cell counts ranged from 0.3 to 3.9 × 10³/µL, with repeated absolute neutrophil counts of 0.0 × 10³/µL, indicating profound neutropenia and severely impaired innate immune defenses. Hemoglobin levels ranged from 6.7 to 9.1 g/dL, while platelet counts fluctuated between 10 and 91 × 10³/µL, necessitating repeated red blood cell and platelet transfusions throughout hospitalization. Peripheral blood differentials demonstrated marked circulating blastemia, with blasts comprising up to 96% of leukocytes, consistent with persistent active leukemia despite ongoing therapy. The combination of severe neutropenia and thrombocytopenia substantially complicated the diagnostic evaluation by increasing susceptibility to opportunistic infections while simultaneously elevating the bleeding risk associated with orbital biopsy, ultimately requiring hematologic optimization before tissue sampling.

**Table 1:** Laboratory findings at presentation and during hospitalization Values are presented as the admission result and the observed range during hospitalization. Hematologic abnormalities reflect relapsed MPAL with chemotherapy-associated marrow suppression during treatment with gilteritinib and venetoclax. Reference ranges correspond to the institutional laboratory standards. ALT, alanine aminotransferase; AST, aspartate aminotransferase; INR, international normalized ratio; MPAL, mixed phenotype acute leukemia.

Laboratory Parameter	At Admission	Range During Hospitalization	Reference Range (Unit)
White blood cell count	0.5	0.3-3.9	4.0-11.0 (×10³/µL)
Absolute neutrophil count	0.0	0.0	1.8-7.7 (×10³/µL)
Hemoglobin	8.3	6.7-9.1	13.5-17.5 (g/dL)
Platelet count	12	10-91	150-450 (×10³/µL)
Peripheral blasts	96%	96%	0%
Sodium	134	134-136	136-145 (mmol/L)
Potassium	3.9	3.6-4.5	3.5-5.1 (mmol/L)
Chloride	104	98-106	98-107 (mmol/L)
Bicarbonate (CO₂)	21	21-24	22-29 (mmol/L)
Blood urea nitrogen	7	6-15	7-20 (mg/dL)
Creatinine	0.60	0.55-0.60	0.70-1.30 (mg/dL)
AST	28	28-43	10-40 (U/L)
ALT	32	31-34	7-56 (U/L)
Alkaline phosphatase	108	108-123	44-147 (U/L)
Total bilirubin	0.5	0.3-0.6	0.2-1.2 (mg/dL)
Prothrombin time	16	16	11-14 (s)
INR	1.26	1.26	0.8-1.2

Serum chemistry studies demonstrated preserved renal function throughout hospitalization, with creatinine values ranging from 0.60 to 0.81 mg/dL. Mild hypoalbuminemia and elevated lactate dehydrogenase levels reflected active hematologic malignancy, increased cellular turnover, and chronic systemic illness. Liver function testing showed only mild intermittent transaminase elevations without evidence of significant hepatic dysfunction (Table 1). Daily laboratory surveillance, including complete blood count, comprehensive metabolic panel, lactate dehydrogenase, uric acid, and phosphorus levels, was performed to monitor disease activity, treatment-related toxicity, and transfusion requirements during continued leukemia-directed therapy.

**Figure 1:**
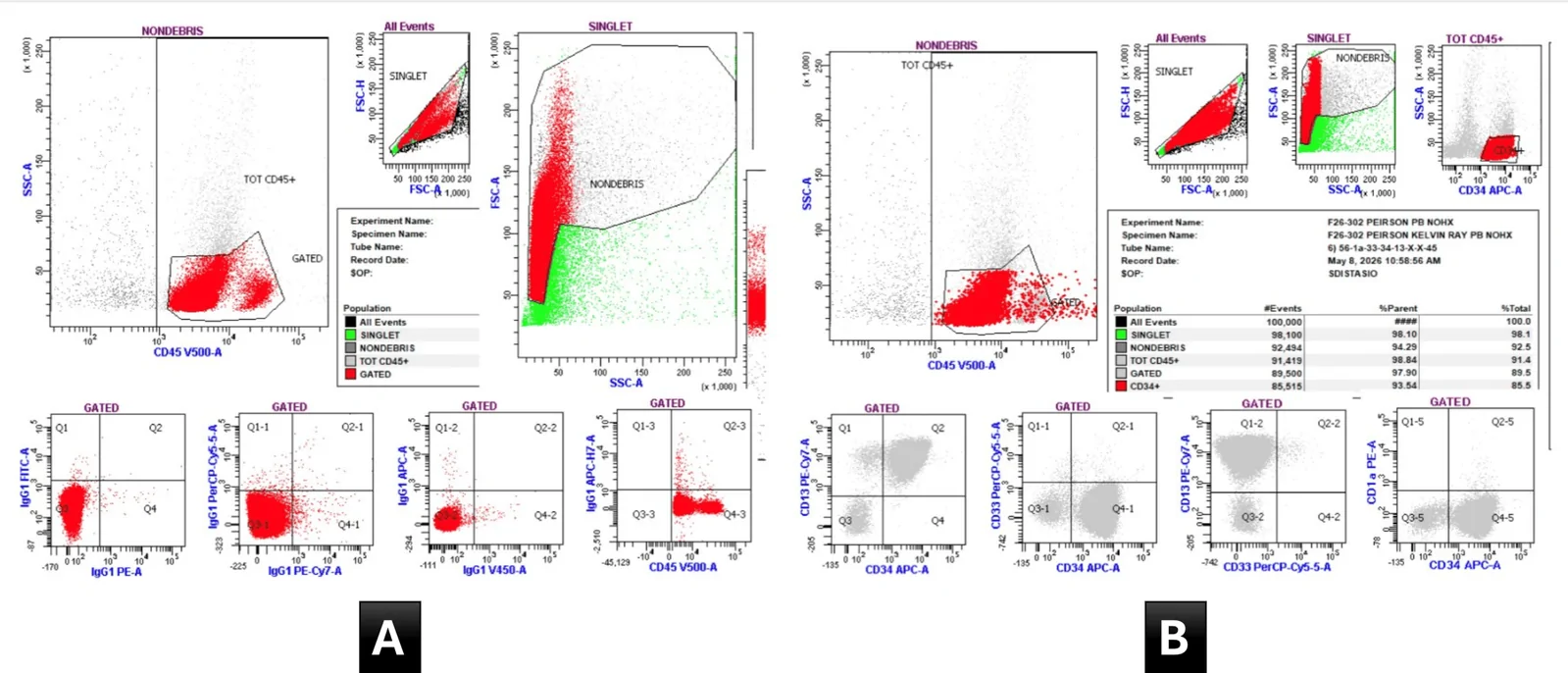
Peripheral blood flow cytometric immunophenotyping demonstrating persistent leukemic blast population (A) Initial gating strategy showing a predominant population of CD45-dim, low side scatter events consistent with circulating immature blasts. (B) Immunophenotypic characterization of the gated blast population demonstrating CD34 positivity with expression of the myeloid-associated antigen CD13 and lack of significant CD33 expression, consistent with persistent active leukemia in the setting of relapsed MPAL. MPAL, mixed phenotype acute leukemia.

**Figure 2:**
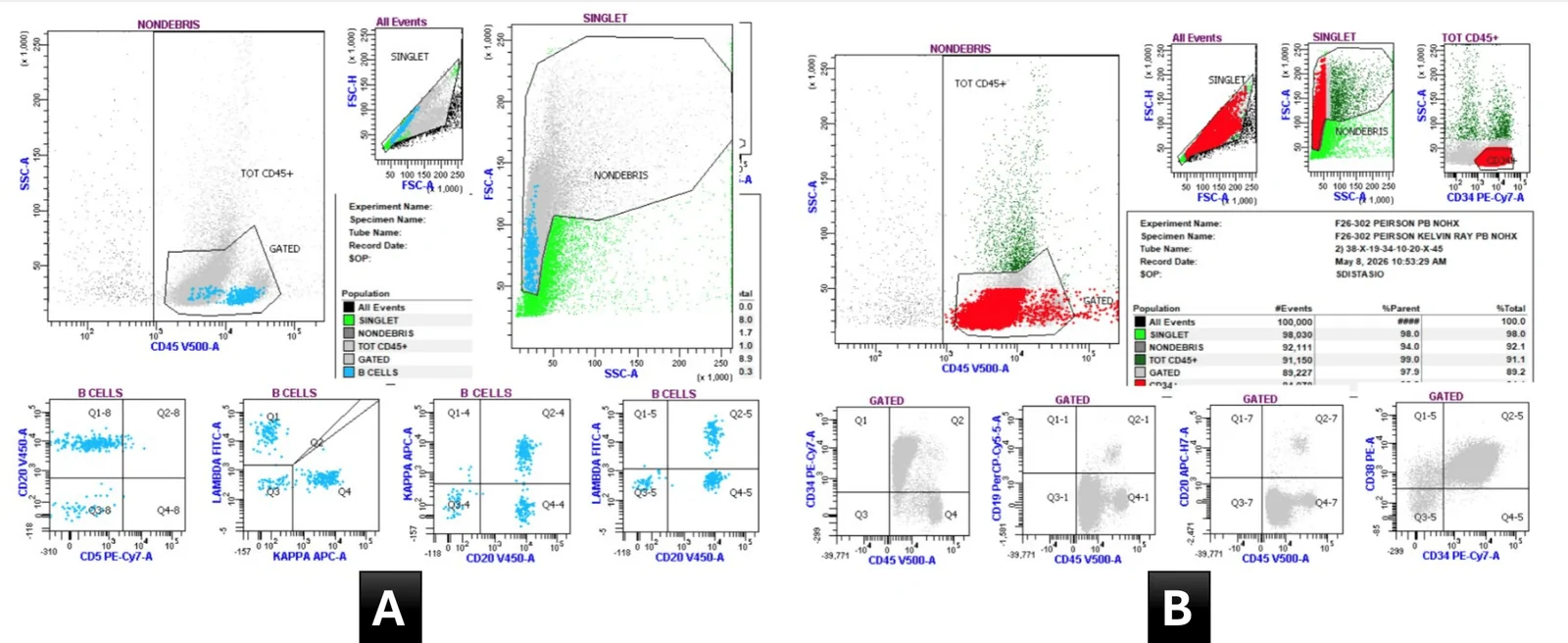
Flow cytometric analysis demonstrating the absence of an abnormal lymphoid population (A) B-cell immunophenotyping demonstrating no evidence of a clonal B-cell population, with no light-chain restriction on kappa/lambda analysis. (B) Additional lymphoid immunophenotyping showing no aberrant B-cell or T-cell immunophenotype. The absence of an abnormal lymphoid population supported exclusion of lymphomatous involvement and was consistent with persistent relapsed mixed phenotype acute leukemia without evidence of a concurrent lymphoid neoplasm.

Peripheral blood flow cytometry demonstrated persistent circulating blasts comprising approximately 80-90% of leukocytes, expressing CD34, CD117, HLA-DR, CD13, and CD33, consistent with relapsed MPAL and persistent circulating disease burden (Figures 1, 2). Peripheral blood smear confirmed marked circulating blastemia and demonstrated significant anisopoikilocytosis with numerous dysmorphic erythrocytes, including tear-drop cells, target cells, schistocytes, elliptocytes, and ovalocytes. These findings were consistent with severe marrow infiltration by relapsed leukemia and treatment-related hematopoietic dysplasia.

**Figure 3:**
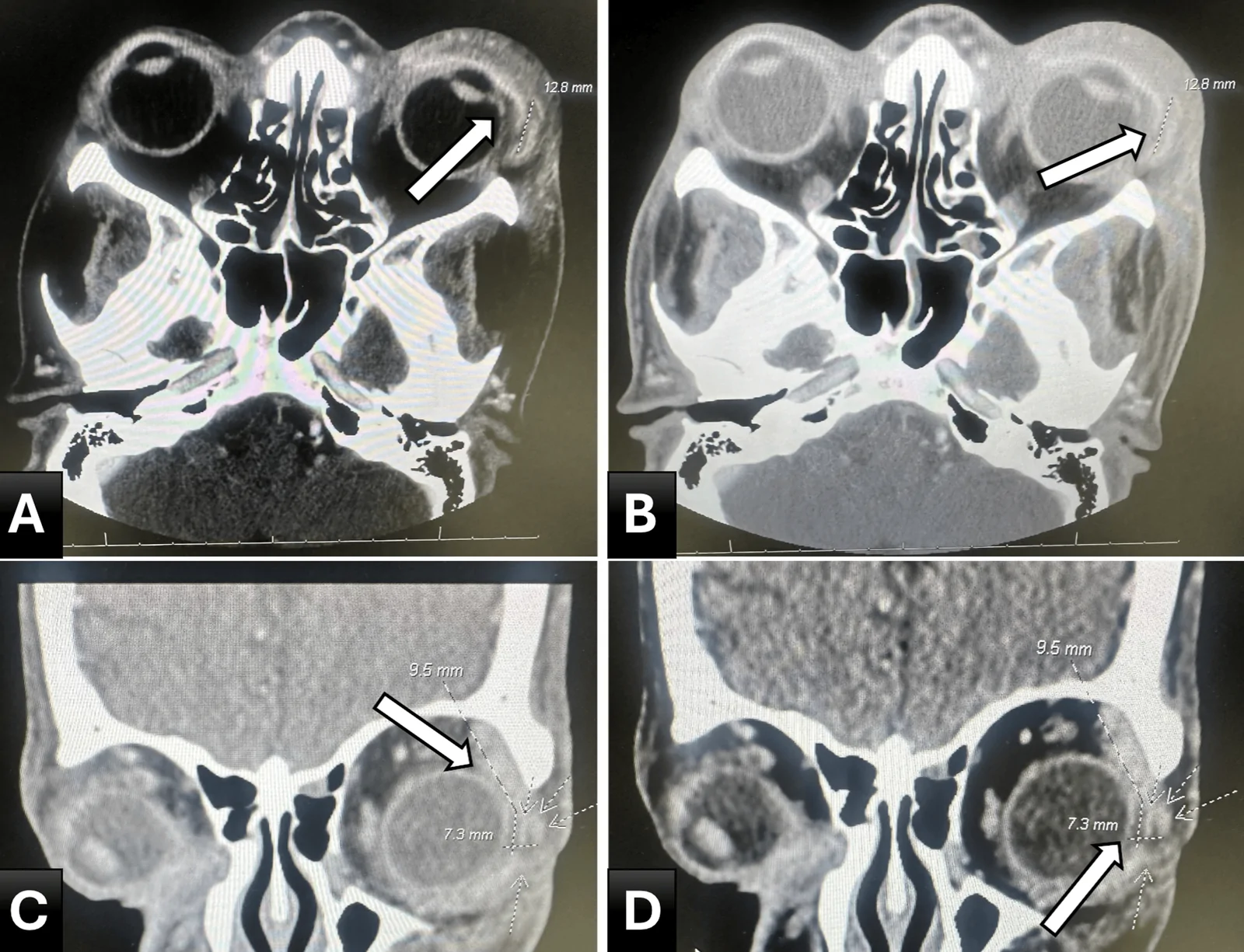
Serial computed tomography imaging of the left orbit demonstrating progressive superior orbital soft tissue thickening (A, B) Initial contrast-enhanced CT of the orbits demonstrates diffuse preseptal and lateral periorbital soft tissue thickening centered within the superolateral left orbit (white arrows), with associated enlargement of the left lacrimal gland and a focal peripherally enhancing hypodense component concerning for evolving phlegmon or abscess. The intraconal fat, extraocular muscles, optic nerve sheath complexes, and orbital osseous structures are preserved. (C, D) Follow-up CT performed after prolonged intravenous antimicrobial therapy demonstrates persistent and mildly progressive soft tissue thickening centered along the temporal aspect of the left orbit (white arrows), with persistent reactive lacrimal gland enlargement and extension into the infraorbital soft tissues despite treatment. The persistence of enhancing orbital soft tissue without a drainable abscess prompted tissue biopsy, which ultimately established invasive conjunctival squamous cell carcinoma.

Given persistent left periorbital swelling despite broad-spectrum antimicrobial therapy, serial cross-sectional imaging was performed to evaluate for progression of infection and alternative etiologies. Initial contrast-enhanced CT demonstrated diffuse preseptal and lateral periorbital soft tissue edema with ill-defined enhancing soft tissue centered within the superior and lateral preseptal orbit and upper eyelid, associated with reactive enlargement of the left lacrimal gland. Importantly, the intraconal fat planes, extraocular muscles, optic nerve sheath complexes, and orbital osseous structures remained preserved without evidence of orbital apex or intracranial extension (Figure 3).

**Figure 4:**
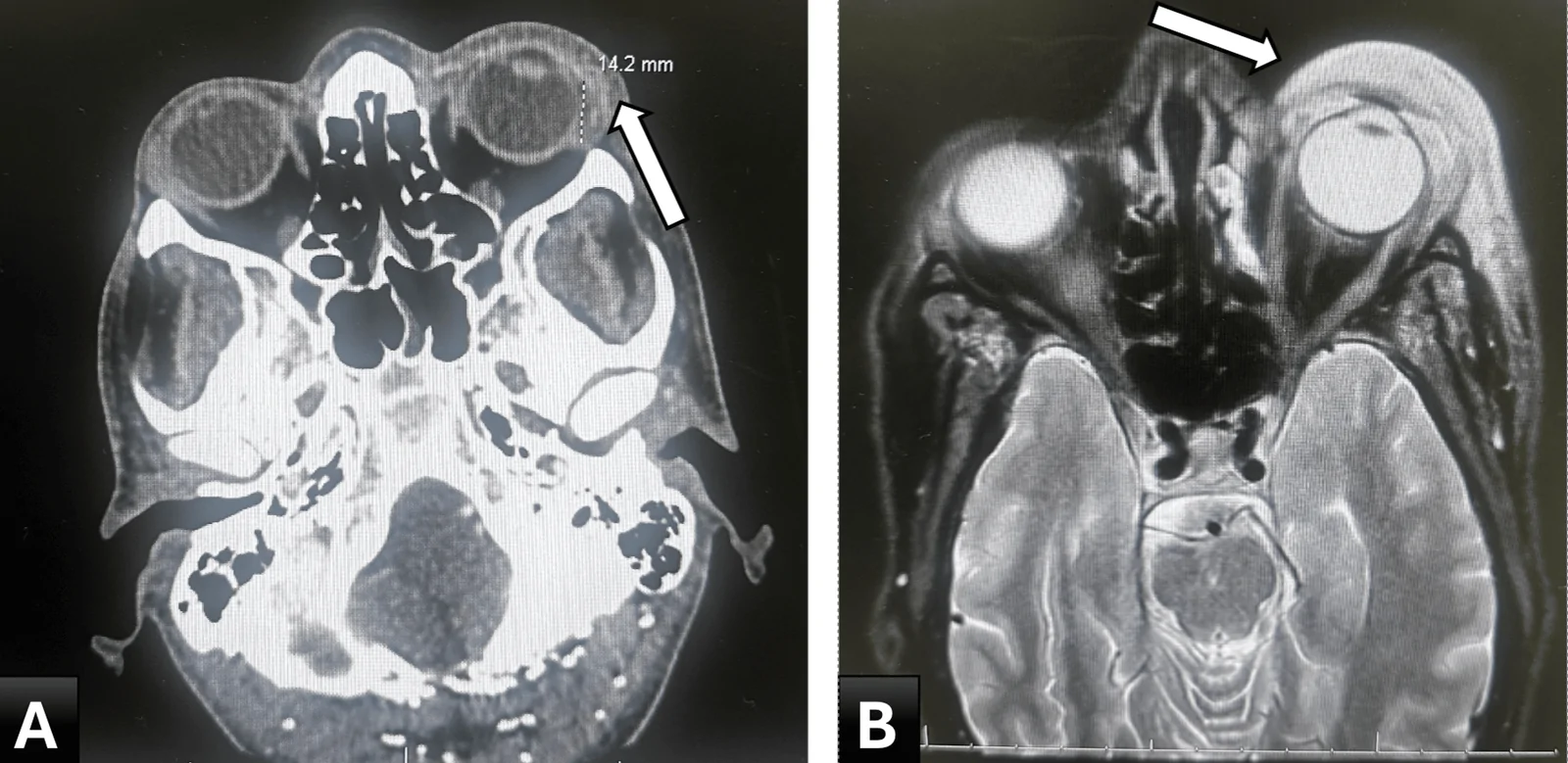
Cross-sectional imaging demonstrating persistent left superior orbital soft tissue thickening (A) Follow-up contrast-enhanced computed tomography (CT) of the orbits demonstrates persistent superolateral left preseptal and periorbital soft tissue thickening (white arrow), measuring up to 14.2 mm, with stable mild proptosis and associated reactive enlargement of the lacrimal gland despite prolonged intravenous antimicrobial therapy. The lesion remained confined to the extraconal compartment without orbital osseous destruction or intraconal extension. (B) Follow-up magnetic resonance imaging (MRI) of the orbits demonstrates persistent abnormal enhancing soft tissue involving the superior left preseptal orbit and palpebral portion of the lacrimal gland (white arrow). The lesion abuts the globe but demonstrates no retrobulbar mass, optic nerve involvement, cavernous sinus extension, or intracranial spread. The persistence of the lesion despite antimicrobial therapy prompted tissue biopsy, which confirmed invasive moderately differentiated conjunctival squamous cell carcinoma.

Repeat CT performed after continued antimicrobial therapy demonstrated persistent and mildly progressive superolateral preseptal soft tissue thickening (12.8 mm to 14.2 mm), stable mild proptosis, persistent reactive dacryoadenitis, enlargement of the superior oblique muscle consistent with reactive myositis, and persistent inferior extraconal fat stranding. Despite these interval changes, there remained no osseous destruction, intraconal invasion, or drainable abscess (Figure 4A).

**Figure 5:**
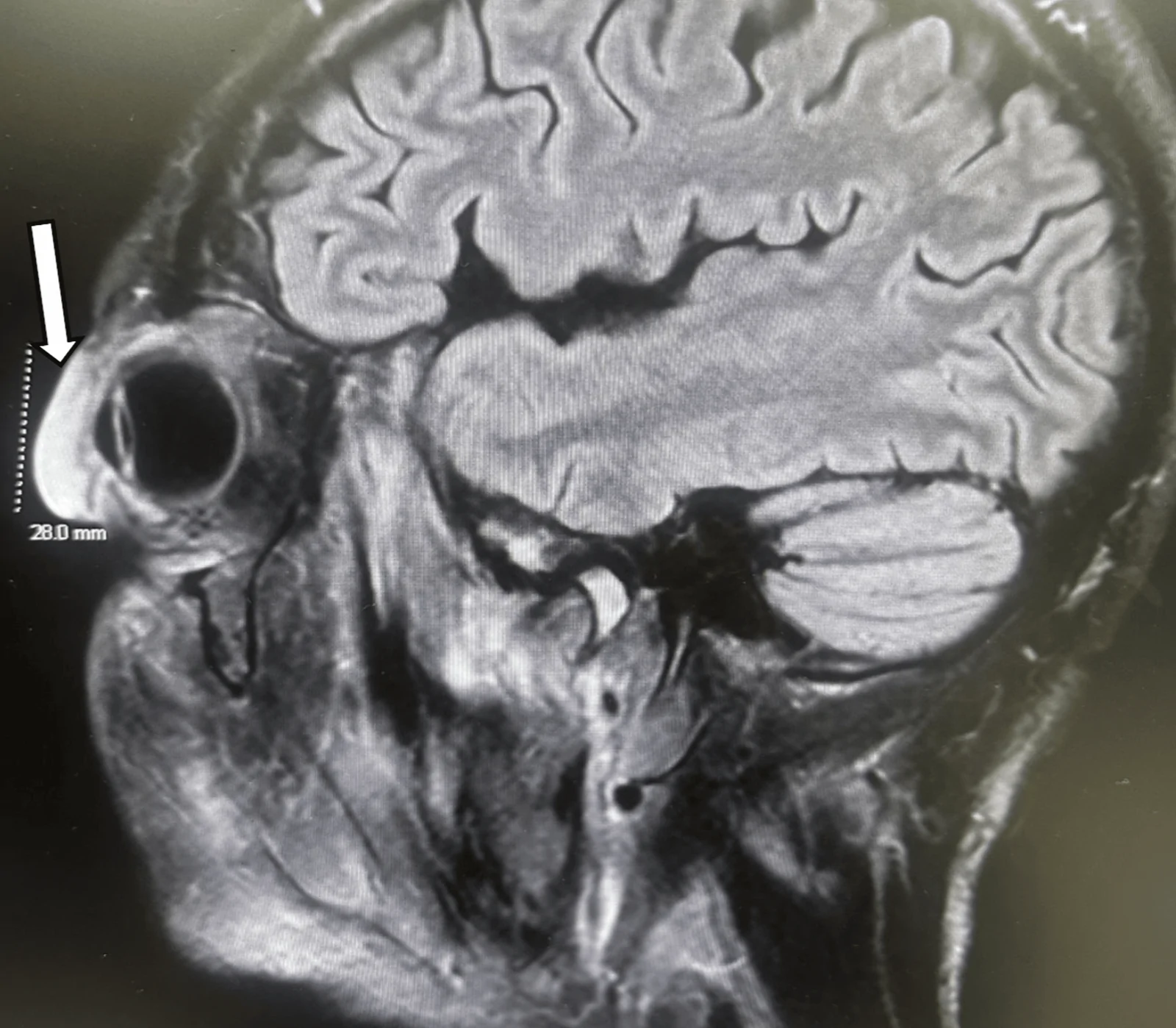
Magnetic resonance imaging of the left orbit demonstrating extensive superior orbital soft tissue involvement Sagittal magnetic resonance image of the left orbit demonstrating marked superior preseptal and orbital soft tissue thickening (arrow) measuring approximately 28.0 mm in maximal dimension. The lesion involves the superior orbital soft tissues adjacent to the lacrimal gland region and results in mass effect upon the superior aspect of the globe. No intracranial extension is identified. Although the imaging appearance was initially interpreted as consistent with an inflammatory or infectious orbital process, subsequent histopathologic examination established the diagnosis of invasive moderately differentiated conjunctival squamous cell carcinoma with lymphovascular invasion.

Follow-up MRI demonstrated persistent enhancing soft tissue inseparable from the palpebral portion of the left lacrimal gland and abutting the globe, with mild edema extending into the adjacent malar soft tissues (Figures 4B, 5). The extraocular muscles, optic nerves, superior ophthalmic veins, cavernous sinuses, and orbital apex remained uninvolved, and no retrobulbar mass or intracranial extension was identified. Although the enhancement was nonspecific and could still represent cellulitis, the persistence of a focal enhancing lesion despite nearly two weeks of broad-spectrum antimicrobial therapy, together with the absence of a drainable abscess and failure of expected radiographic resolution, raised concern for an alternative infiltrative process and prompted tissue biopsy, which established the diagnosis of invasive conjunctival SCC.

Concurrent evaluation of gross hematuria demonstrated BK polyomavirus reactivation, supporting a diagnosis of BK virus-associated hemorrhagic cystitis. Computed tomography of the abdomen and pelvis and urologic evaluation revealed no structural urinary tract abnormalities.

Because of persistent orbital findings despite extensive antimicrobial treatment, tissue diagnosis was pursued. Histopathologic examination of the left conjunctival lesion revealed invasive moderately differentiated SCC with lymphovascular invasion. Flow cytometric analysis of the biopsy specimen demonstrated no abnormal B-cell or T-cell populations, excluding lymphomatous involvement and supporting a primary epithelial malignancy rather than leukemic infiltration.

Multidisciplinary board discussion

The patient's case was reviewed by a multidisciplinary team including hematology-oncology, ophthalmology, oculoplastics, pathology, infectious disease, radiology, and radiation oncology. The primary challenge was determining whether the persistent orbital lesion represented refractory infection, leukemic infiltration, opportunistic infection, or a secondary malignancy in the setting of relapsed MPAL and profound immunosuppression.

Although initial clinical and radiographic findings favored orbital cellulitis, the lesion failed to resolve despite prolonged broad-spectrum antimicrobial therapy. Persistent orbital soft tissue thickening on serial imaging prompted tissue diagnosis. Biopsy report of the left upper eyelid/conjunctival lesion confirmed SCC as the primary cause of the persistent orbital abnormality.

Given the patient's active relapsed leukemia, ongoing chemotherapy, severe cytopenias, and transfusion dependence, the multidisciplinary team recommended continued supportive care and outpatient ophthalmologic, oncologic, and radiation oncology follow-up for definitive treatment planning.

Diagnosis and management

The patient's persistent unilateral orbital inflammation despite prolonged broad-spectrum intravenous antimicrobial therapy, together with serial imaging demonstrating a persistent enhancing soft tissue lesion without a drainable abscess, prompted ophthalmology and oculoplastic surgery to obtain tissue for histopathologic diagnosis. Because the patient had profound chemotherapy-associated thrombocytopenia requiring ongoing transfusion support, biopsy was performed only after careful multidisciplinary discussion and peri-procedural hematologic optimization to minimize bleeding risk.

Histopathologic examination of the left upper eyelid conjunctival lesion demonstrated invasive moderately differentiated SCC with lymphovascular invasion, establishing the diagnosis of invasive ocular surface SCC (OSSN) with orbital extension. Flow cytometric analysis of the biopsy specimen demonstrated no abnormal B-cell or T-cell populations, excluding lymphomatous involvement and making leukemic chloroma unlikely.

Following establishment of the diagnosis, management was coordinated among ophthalmology, oculoplastic surgery, hematology-oncology, infectious diseases, and radiation oncology. Broad-spectrum antimicrobial therapy was subsequently de-escalated after malignancy became the primary explanation for the persistent orbital lesion, while supportive antimicrobial prophylaxis was continued because of persistent profound neutropenia. Leukemia-directed therapy with gilteritinib and venetoclax was continued under close hematologic supervision because uncontrolled relapsed MPAL remained the patient's immediately life-threatening condition. Definitive local treatment of the conjunctival SCC was deferred until stabilization of the patient's hematologic status and completion of multidisciplinary treatment planning, given the substantial procedural risk posed by ongoing marrow failure and transfusion-dependent cytopenias.

The coexistence of relapsed MPAL and invasive conjunctival SCC created a significant therapeutic dilemma. Profound neutropenia and thrombocytopenia increased the risks of surgical intervention, impaired wound healing, and heightened susceptibility to infectious complications, while interruption of leukemia-directed therapy carried the risk of rapid hematologic progression. In addition, prolonged immunosuppression resulting from relapsed leukemia together with treatment using venetoclax, a BCL-2 inhibitor, and gilteritinib, an FLT3 inhibitor, likely contributed to impaired immune surveillance, creating a biologic environment that may facilitate the development and aggressive behavior of secondary epithelial malignancies. Although a direct causal relationship cannot be established from a single case, this clinical scenario highlights the importance of maintaining suspicion for secondary solid tumors in profoundly immunocompromised patients whose presumed infections fail to respond as expected.

Clinical outcome and follow-up 

Following histopathologic confirmation of invasive moderately differentiated conjunctival SCC with lymphovascular invasion, the patient remained clinically stable without evidence of visual deterioration, orbital apex involvement, or intracranial extension during the remainder of his hospitalization. Although the surrounding periorbital inflammatory changes improved, the focal orbital lesion persisted despite prolonged intravenous antimicrobial therapy, supporting the diagnosis of an underlying malignancy rather than uncomplicated orbital cellulitis.

The patient was discharged with coordinated outpatient follow-up involving hematology-oncology, ophthalmology, oculoplastic surgery, and radiation oncology for continued management of relapsed MPAL and invasive conjunctival SCC. Because of profound transfusion-dependent pancytopenia and active leukemia, definitive treatment of the ocular malignancy was deferred pending multidisciplinary outpatient evaluation. Long-term follow-up, including subsequent oncologic treatment, disease progression, and survival outcomes, was unavailable.

## Discussion

Background 

OSSN comprises a spectrum of epithelial lesions ranging from conjunctival intraepithelial neoplasia to invasive SCC and represents the most common non-pigmented malignancy of the ocular surface [3,4]. The entity was first recognized as a distinct clinicopathologic process in the mid-20th century, and advances in histopathologic classification have since improved understanding of its biologic behavior and malignant potential [4]. According to the World Health Organization (WHO) classification, OSSN encompasses dysplastic, preinvasive, and invasive squamous epithelial lesions arising from the conjunctival and corneal epithelium [8].

The incidence of OSSN varies geographically, with the highest rates reported in regions with intense ultraviolet (UV) radiation exposure, particularly equatorial Africa and Australia [9]. The disease most commonly affects older adults and demonstrates a male predominance, although younger patients may be affected in the presence of significant immunosuppression [5,9]. Established risk factors include chronic UV-B exposure, human papillomavirus infection, human immunodeficiency virus infection, cigarette smoking, xeroderma pigmentosum, and conditions associated with impaired immune surveillance [4,5,9,10].

Conjunctival SCC most frequently arises at the limbus within the interpalpebral fissure, where ultraviolet exposure is greatest [3,5]. While many lesions remain localized and are associated with favorable outcomes following surgical excision and adjuvant therapy, invasive tumors may extend into the globe, orbit, or adjacent structures and can exhibit lymphovascular invasion and regional metastasis [5,11]. Orbital extension is uncommon but represents a more aggressive disease phenotype associated with increased morbidity [11].

Orbital cellulitis remains the most common cause of acute orbital inflammation; however, inflammatory, infiltrative, and neoplastic disorders may closely mimic its clinical and radiographic appearance [2]. In immunocompromised patients, distinguishing infection from malignancy can be particularly challenging, emphasizing the importance of maintaining a broad differential diagnosis and obtaining tissue diagnosis when the clinical course is atypical or treatment response is incomplete [2,12].

Physiology/pathophysiology 

Conjunctival SCC develops through progressive genetic and molecular alterations that disrupt normal epithelial cell-cycle regulation and apoptosis, most commonly following chronic ultraviolet-B (UV-B)-induced DNA damage [6,10]. Progression from dysplasia to invasive disease requires disruption of the epithelial basement membrane and acquisition of an invasive phenotype, processes that are normally constrained by effective immune surveillance [6,7,9].

In this patient, relapsed MPAL, profound marrow failure, and prolonged treatment-related immunosuppression likely created a permissive environment for aggressive tumor behavior. Patients with hematologic malignancies experience impaired immune surveillance, predisposing them to opportunistic infections and secondary malignancies while reducing the clearance of dysplastic epithelial cells [6,7,9]. This immune dysfunction may have facilitated rapid local invasion and the lymphovascular invasion observed on histopathology [7,13,14].

The pseudo-infectious presentation can be explained by tumor-associated inflammation. Local invasion of the conjunctiva and adjacent orbital tissues produces edema, erythema, pain, and reactive inflammation that may closely mimic orbital cellulitis both clinically and radiographically [7,11,12,15]. In this case, persistent orbital inflammation despite prolonged broad-spectrum antimicrobial therapy reflected malignant infiltration rather than uncontrolled infection, emphasizing the importance of tissue biopsy when presumed orbital cellulitis fails to respond as expected [2,11,12].

Comparative analysis/literature review****

Conjunctival SCC typically presents as a visible limbal or bulbar conjunctival lesion with a gelatinous, leukoplakic, papilliform, or nodular appearance and is usually confined to the ocular surface at diagnosis [7,11,14]. Orbital cellulitis, in contrast, commonly presents with acute periorbital pain, erythema, eyelid edema, fever, ophthalmoplegia, and inflammatory radiographic findings requiring prompt antimicrobial therapy [1,2,15].

Our patient instead presented with progressive periorbital swelling, erythema, pain, dacryoadenitis, and imaging findings highly suggestive of orbital cellulitis despite the absence of a classic ocular surface mass, creating a significant diagnostic challenge. Similar atypical presentations have been reported in immunocompromised patients, in whom impaired immune surveillance is associated with more aggressive OSSN and a higher likelihood of invasive disease [9,13,16].

This case also highlights the limitations of orbital imaging in differentiating infection from malignancy [11,12,15]. Computed tomography and magnetic resonance imaging demonstrated preseptal soft tissue thickening, lacrimal gland enlargement, inflammatory changes, and mild proptosis, findings commonly encountered in orbital cellulitis and inflammatory orbital disease [2,12,15]. In retrospect, however, several imaging features were less consistent with uncomplicated infection, including persistent focal soft tissue enlargement, preservation of the intraconal compartment, absence of osseous destruction, and failure of the lesion to regress despite prolonged broad-spectrum antimicrobial therapy [11,12,15]. Although none of these findings is individually diagnostic of malignancy, persistent focal orbital abnormalities despite appropriate antimicrobial therapy should prompt reconsideration of the differential diagnosis and early tissue biopsy [11,12].

Current management guidelines for OSSN recommend complete surgical excision with histopathologic confirmation for suspected invasive disease, while persistent or atypical orbital inflammation warrants biopsy when the clinical course is inconsistent with infection [11,13,14,17,18]. In our patient, profound pancytopenia, absolute neutropenia, transfusion-dependent thrombocytopenia, and active relapsed MPAL substantially complicated this decision by increasing procedural risk while simultaneously making delayed diagnosis potentially detrimental [3,11]. Despite these challenges, ophthalmologic biopsy established the diagnosis of invasive moderately differentiated conjunctival SCC with lymphovascular invasion, and concurrent flow cytometry excluded lymphomatous involvement, confirming a second primary epithelial malignancy rather than leukemic infiltration.

Published literature provides limited guidance regarding the diagnosis and management of conjunctival SCC occurring concurrently with relapsed MPAL and severe treatment-related marrow failure [3,11,13,14]. This case expands the existing literature by demonstrating that persistent orbital inflammation in profoundly immunocompromised patients should not be attributed to infection alone [11-13]. When clinical response and serial imaging remain discordant despite appropriate antimicrobial therapy, early multidisciplinary reassessment and tissue diagnosis are essential to avoid delays in diagnosing an underlying malignancy [11-14]. Table 2 summarizes the comparative analysis of this case with the current literature. 

**Table 2:** Comparison of typical orbital cellulitis, typical conjunctival squamous cell carcinoma, and the present case MPAL, mixed phenotype acute leukemia; SCC, squamous cell carcinoma; UV, ultraviolet.

Feature	Typical Orbital Cellulitis	Typical Conjunctival SCC	Present Case
Patient population	Any age; often associated with sinusitis or local infection [1,2].	Older adults; risk increased by UV exposure and immunosuppression [9,16,18].	40-year-old man with relapsed MPAL, profound pancytopenia, and prolonged immunosuppression.
Typical presentation	Acute periorbital pain, erythema, eyelid edema, fever, ophthalmoplegia [1,2,14].	Visible limbal/bulbar conjunctival mass, often gelatinous, leukoplakic, or papilliform [5,11,18].	Progressive periorbital swelling, erythema, pain, dacryoadenitis, without a classic ocular surface lesion.
Imaging findings	Preseptal/postseptal edema, inflammatory fat stranding, abscess or phlegmon may be present [2,14].	Localized conjunctival mass with possible orbital extension in advanced disease [11,16,18].	Persistent superior orbital soft tissue thickening, lacrimal gland enlargement, mild proptosis, preserved intraconal compartment, no osseous destruction.
Response to antimicrobial therapy	Clinical and radiographic improvement expected with appropriate treatment [1,2].	No meaningful response to antimicrobial therapy [11,18].	Persistent orbital lesion despite prolonged broad-spectrum antimicrobial therapy.
Definitive diagnosis	Clinical evaluation supported by imaging; cultures when indicated [1,2].	Histopathologic examination of biopsy or excision specimen [11,15,18].	Ophthalmologic biopsy confirmed invasive moderately differentiated conjunctival SCC with lymphovascular invasion.
Major diagnostic challenge	Distinguishing infection from inflammatory or neoplastic orbital disease [12,14].	Advanced tumors may masquerade as orbital inflammation or cellulitis [11,16,18].	Profound immunosuppression and imaging findings strongly favored infection, delaying recognition of an underlying malignancy.
Key clinical lesson	Failure to improve should prompt reassessment of the diagnosis [2,12].	Early tissue diagnosis is recommended when invasive disease is suspected [11,15,18].	Persistent orbital inflammation despite appropriate therapy should prompt early biopsy, even in profoundly immunocompromised patients.

What we learned from this case 

This case demonstrates that invasive conjunctival SCC can closely mimic orbital cellulitis in profoundly immunocompromised patients with hematologic malignancies, making diagnosis particularly challenging when clinical presentation and radiographic findings strongly favor infection. Although orbital cellulitis remains the most likely diagnosis in neutropenic patients presenting with fever, periorbital swelling, and imaging consistent with orbital inflammation, persistent or only partially resolving orbital abnormalities despite appropriate antimicrobial therapy should prompt early reassessment of the working diagnosis and consideration of an underlying neoplastic process.

A second important lesson is that imaging alone cannot reliably distinguish invasive conjunctival SCC from infectious orbital disease. In our patient, serial imaging continued to demonstrate orbital inflammation despite treatment, and definitive diagnosis was established only after histopathologic examination. This reinforces the central role of tissue diagnosis when the clinical course is inconsistent with the expected response to therapy.

This case also illustrates the complexity of clinical decision-making in patients with profound treatment-related cytopenias. While timely biopsy is essential for establishing a definitive diagnosis, the procedure must be balanced against the risk of bleeding and other procedure-related complications in patients with severe thrombocytopenia or marrow failure. Our patient's biopsy was therefore appropriately delayed until hematologic optimization allowed tissue sampling to be performed more safely.

Finally, whether the conjunctival SCC represented an incidental second primary malignancy or developed in association with prolonged immunosuppression and cumulative leukemia-directed therapy remains uncertain. Although an increased incidence of secondary solid malignancies has been reported following treatment for hematologic cancers, OSSN has rarely been described in this setting. Additional studies are needed to clarify the relationship between prolonged leukemia-directed therapy, immune dysfunction, and secondary ocular malignancies.

Limitations

This report has several limitations inherent to a single-patient case study. First, the rarity of concurrent relapsed MPAL and invasive conjunctival SCC limits the generalizability of our observations and precludes establishing causal relationships between profound immunosuppression and tumor development. Second, although imaging findings were reviewed retrospectively, radiographic features alone could not reliably distinguish malignancy from orbital cellulitis, emphasizing that definitive diagnosis depended on histopathologic examination rather than imaging characteristics. Third, ancillary molecular analyses, including human papillomavirus (HPV) and Epstein-Barr virus (EBV) testing of the tumor, were not performed; therefore, the contribution of viral oncogenic factors could not be assessed. Finally, long-term oncologic follow-up was unavailable, preventing evaluation of treatment response, recurrence, disease progression, and overall survival.

Despite these limitations, the present case documents an exceptionally uncommon clinical presentation in which invasive conjunctival SCC masqueraded as orbital cellulitis in a profoundly immunocompromised patient with relapsed MPAL. The case underscores the importance of maintaining a broad differential diagnosis and pursuing timely tissue biopsy when orbital inflammation persists despite appropriate antimicrobial therapy, particularly in patients with underlying hematologic malignancies.

## Conclusions

This case highlights an uncommon but clinically important diagnostic challenge in which invasive conjunctival SCC masqueraded as orbital cellulitis in a patient with relapsed MPAL. Despite appropriate antimicrobial therapy, persistent orbital abnormalities warranted reassessment of the diagnosis, ultimately leading to tissue biopsy and definitive histopathologic confirmation of malignancy. This case reinforces the limitations of clinical presentation and imaging alone in distinguishing infectious from neoplastic orbital processes in profoundly immunocompromised patients.

The decision to pursue tissue biopsy should be individualized, balancing the urgency of establishing a definitive diagnosis against procedure-related risks, particularly in patients with severe thrombocytopenia or other hematologic contraindications. Although it remains uncertain whether the conjunctival SCC represented an incidental second primary malignancy or developed in association with prolonged immunosuppression and cumulative leukemia-directed therapy, this case raises important questions regarding the pathogenesis of secondary ocular malignancies in patients with hematologic cancers. Greater awareness of this rare presentation may facilitate earlier recognition of occult orbital malignancy and help reduce diagnostic delays in similarly complex patients.
